# Supplementary material for: Unveiling the impact of cGMP-dependent protein kinase of Neospora caninum on calcium fluxes and egress functions through quantitative phosphoproteome analysis
Source: Commun Biol. 2025 May 13;8:744. doi: 10.1038/s42003-025-08173-x (PMC12075863; doi:10.1038/s42003-025-08173-x)
Supplement: Supplementary file 2 — Supplementary Information [file 42003_2025_8173_MOESM2_ESM.pdf]

# Unveiling the Impact of cGMP-Dependent Protein Kinase of *Neospora caninum* on Calcium Fluxes and Egress Functions Through Quantitative Phosphoproteome Analysis

Xianmei Wang[1], Kun Guo[1], Zhili Shan[1], Zhu Ying[1], Zifu Zhu[1], Shiman Yang[1], Na Yang[1], Qun Liu[1,2], Lifang Wang[1], Jing Liu[1,2*]

[1]National Animal Protozoa Laboratory, College of Veterinary Medicine, China Agricultural University, Beijing 100193, PR China.

[2] National Key Laboratory of Veterinary Public Health and Safety, Key Laboratory of Animal Epidemiology of Ministry of Agriculture and Rural Affairs, College of Veterinary Medicine, China Agricultural University, Beijing 100193, PR China.

Corresponding author: Jing Liu

E-mail: liujingvet@cau.edu.cn

**This PDF file includes:**
**Supplementary Figures 1 to 6**
**Supplementary Data 1 Sequence information**

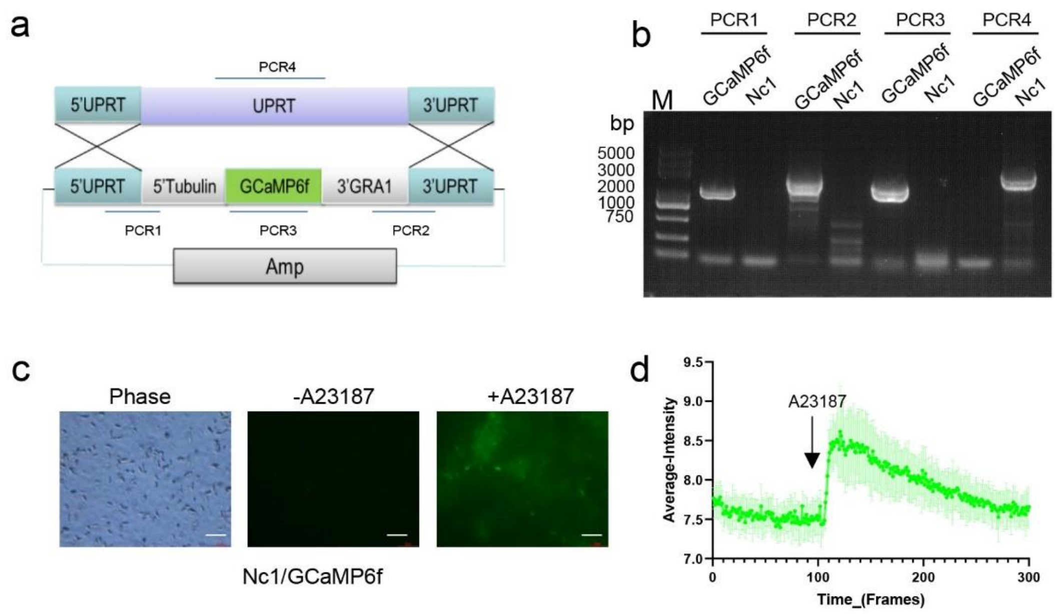


**Supplementary Figure 1 Construction and identification of the calcium indicator Nc1/GCaMP6f strain in *N. caninum*. a** Schematic representation of the construction of Nc1/GGamP6f strain. The NcUPRT gene was replaced with GCaMP6f and expressed by the Tubulin promoter. **b** PCR confirmation of successful 5' and 3'-homologous recombination in Nc1/GGamP6f monoclonal strains. The amplified fragments are shown in panel (**a**). **c** Fluorescence detection of Nc1/GGamP6f strains. Images from left to right: phase contrast, resting state fluorescence, and fluorescence post-A23187 application. Scale bars: 5 μm. **d** Real-time monitoring of fluorescence intensity changes in Nc1/GGamP6f parasites following A23187 stimulation.

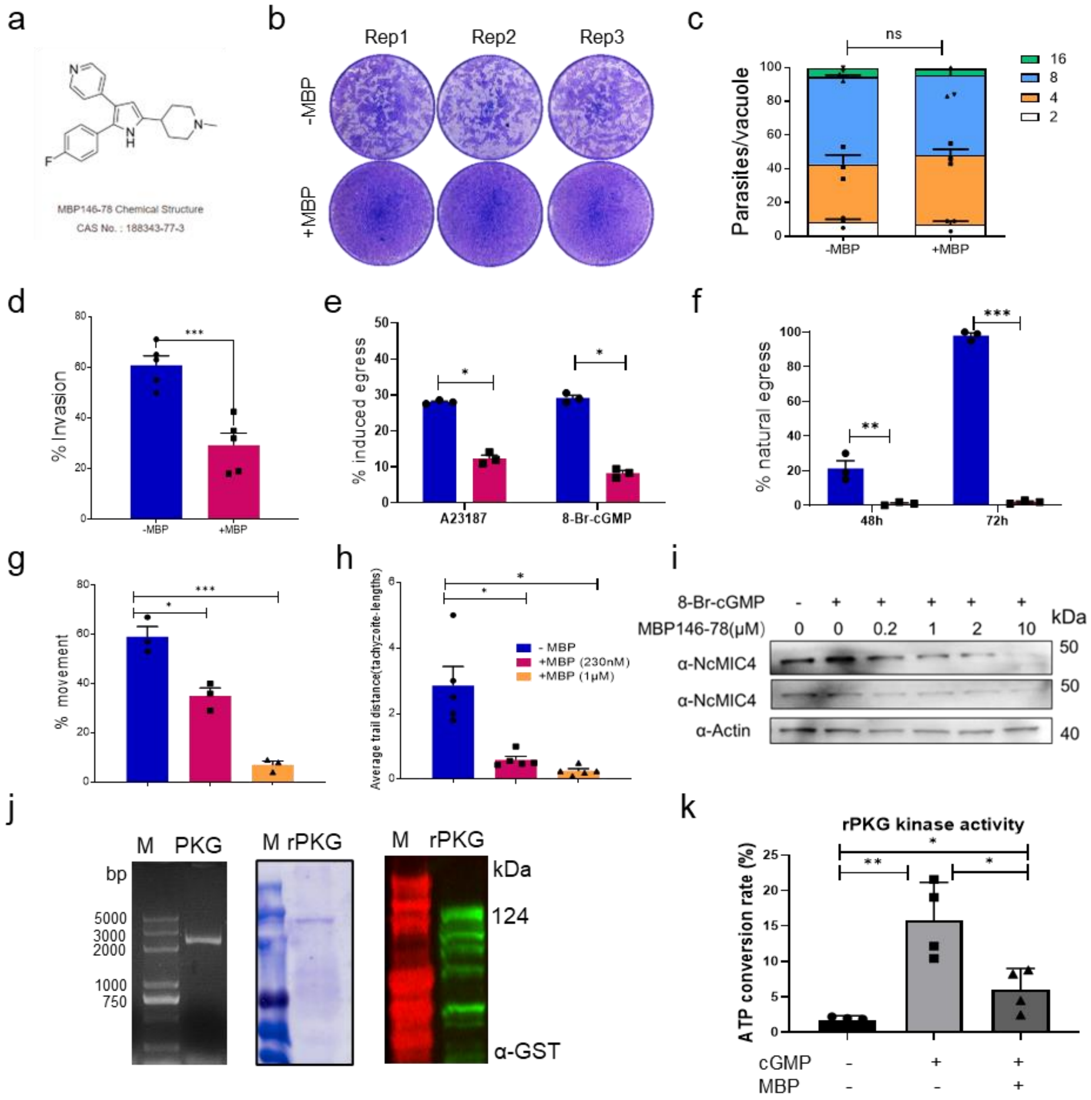


**Supplementary Figure 2 Functional analysis of compound MBP146-78 in the lytic cycle of *N. caninum*. a** Molecular structure of compound MBP146-78. **b** Inhibitory effect of MBP146-78 on *N. caninum*. Plaque formation of *N. caninum* in HFF cells with or without MBP146-78. **c** Comparison of intracellular proliferation in the Nc1 strain with or without MBP146-78 (230 nM) treatment for 30 hours. The mean and standard error of the mean are shown, from three experiments; ns, not significant. **d** Invasion efficiency of Nc1 strain with or without MBP146-78 (230 nM) treatment. Tachyzoites were precultured with or without MBP146-78 (230 nM) for 2

hours and then allowed to invade host cells for 1 hour. **e** Egress efficiency of Nc1 strain with or without MBP146-78 (230 nM) treatment. Tachyzoites were precultured with or without compound for 2 hour and then induced with calcium ionophore A23187 (1 μM) and 8-Br-cGMP (5 μM) for 3 minutes. **f** Proportion of naturally egressed parasites in Nc1 strain with or without MBP146-78 (230 nM) treatment for 48 hours and 72 hours. **g-h** Motility assay of Nc1 strain with or without MBP146-78 (230 nM) treatment. Tachyzoites were precultured with or without MBP146-78 (230 nM or 1 μM) for 2 hour and then placed on cell slides. Movement was induced with 5 μM 8-Br-cGMP. The proportion of motile tachyzoites **g** and the movement trail distance in tachyzoite-lengths **h** were measured. **d-h** The mean and standard error of the mean are shown from three experiments; *$p< 0.05$, **$p< 0.01$, ***$p< 0.001$, ****$p< 0.0001$, ns, not significant (unpaired two-tailed Student's t test). **i** Microneme secretion in the Nc1 strain with or without MBP146-78 (230 nM) treatment. Tachyzoites were precultured with different concentrations of MBP146-78 for 2 hours, then induced with 5 μM 8-Br-cGMP for 15 minutes. Western blotting analyzed microneme content in supernatant ESA (top) and pellet (middle). Antibodies α-MIC4 were used for the detection of microneme protein 4 expression and secretion, and α-actin was used as a loading control. **j** Expression and identification of recombinant proteins. The PCR image (left) shows amplification of the 2,541 bp NcPKG gene fragments. The middle SDS-PAGE image shows the purified rPKG (~124 kDa) using a glutathione column. Western blotting (right) confirmed recombinant expression using a GST antibody, detecting the GST-tagged protein at ~124 kDa; additional

bands indicate possible recombinant protein degradation. **k** Protein rNcPKG kinase activity assay. From left to right: reaction system without activator cGMP, with cGMP, and with both cGMP and MBP146-78 inhibitor. The mean and standard error of the mean are shown from three experiments; **p*< 0.05, ***p*< 0.01, ****p*< 0.001, *****p*< 0.0001, ns, not significant (unpaired two-tailed Student's t test).

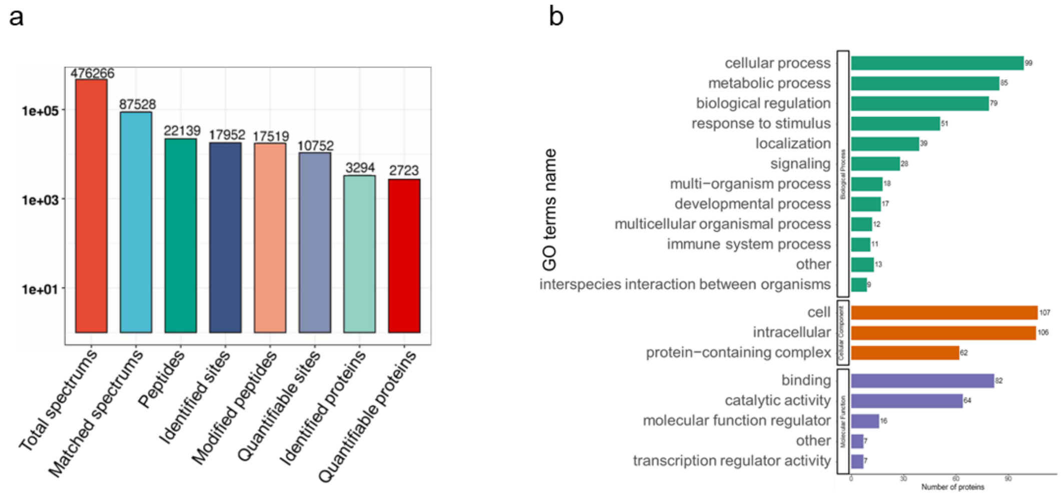


**Supplementary Figure 3 Phosphorylation quantitative omics and GO function analysis of PKG-regulated downstream molecules. a** Overview of protein identification by phosphorylation quantitative omics. A total of 17,519 phosphorylated peptide sequences and 3,294 phosphorylated proteins were identified in *N. caninum*. **b** GO analysis of potential downstream phosphorylated proteins regulated by PKG. Proteins were classified according to biological process, cellular component, and molecular function. Molecular functions include binding activity, catalytic activity, regulation of molecular functions, transcriptional regulatory activity, and other important functions.

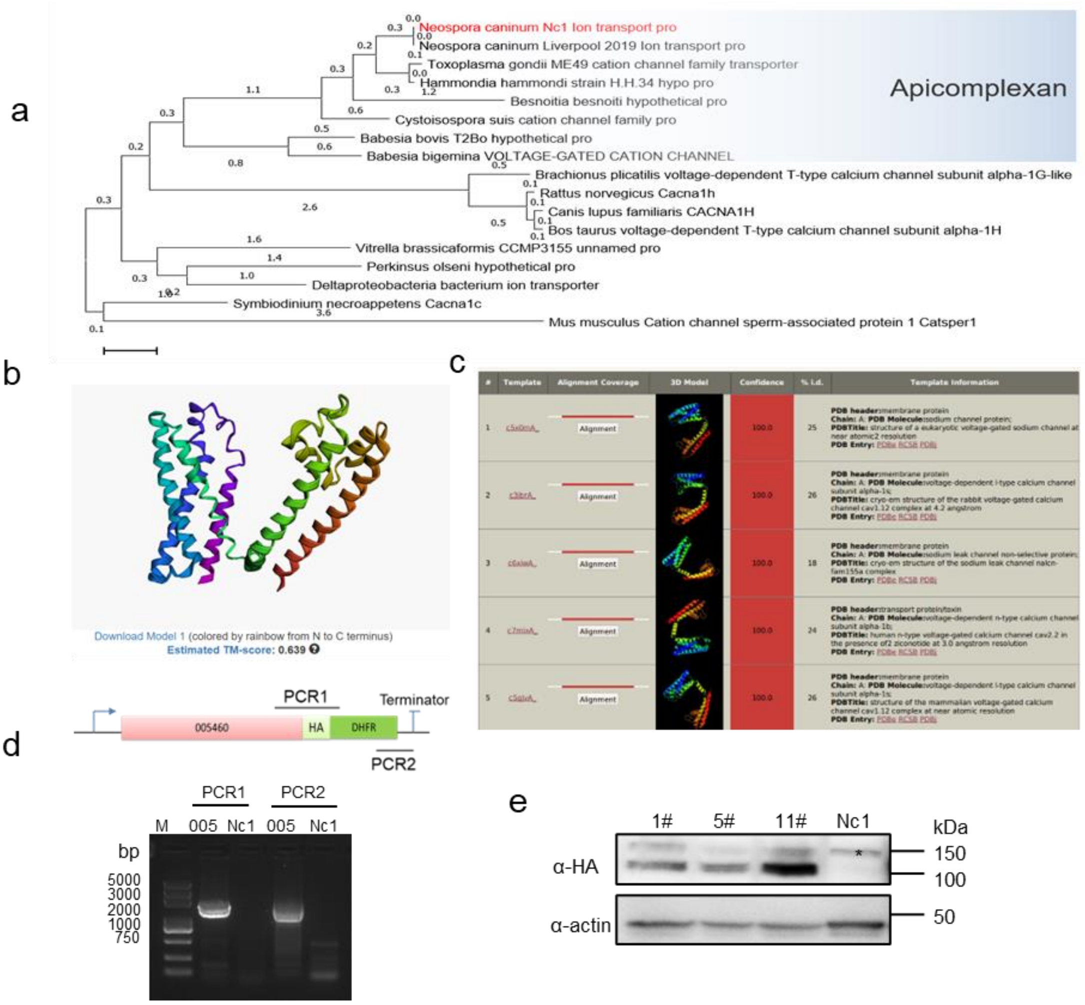


**Supplementary Figure 4 Identification of CACNAP in *N. caninum*. a** Evolutionary analysis of proteins homologous to NCLIV_005460. **b** Advanced structural modeling of ion transport domains in NCLIV_005460. **c** Protein structures similar to the ion transport domain of NCLIV_005460. **d** Strategy for construction of CACNAP endogenous HA strains with PCR verification of correct homologous recombination. **e** Western blotting detected CACNAP expression in *cacnap*-HA parasites using α-HA as the primary antibody for HA-tagged fusion proteins. α-actin was used as a loading control. Bands marked with * indicate non-specific proteins.

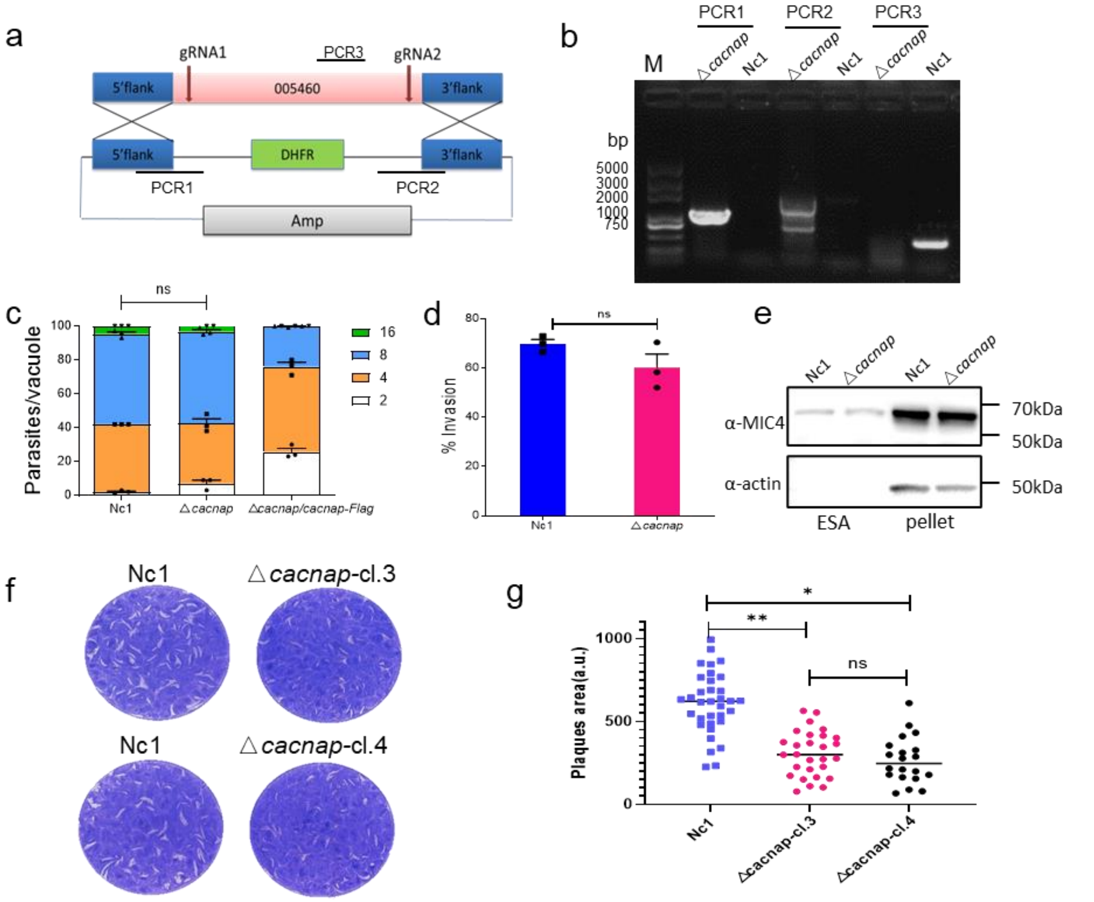


**Supplementary Figure 5 Knockout of CACNAP in *N. caninum* and phenotypic analysis of △*cacnap* strains. a** Schematic of the construction of △*cacnap* strain. The CACNAP gene was replaced by DHFR. **b** PCR confirmation of correct 5' and 3' homologous recombination in △*cacnap* monoclonal strains. The amplified fragments are shown in (**a**). **c** Comparison of intracellular proliferation between Nc1 and △*cacnap* strains for 30 hours. The mean and standard error of the mean from three experiments are shown, ns, not significant. **d** Invasion efficiency statistics for Nc1 and △*cacnap* strains. Tachyzoites were collected to invade host cells for 1 hour. The mean and standard error of the mean from three experiments are shown, ns, not significant (unpaired two-tailed Student t test). **e** Microneme secretion in Nc1 and △

*cacnap* strains. Tachyzoites were collected and induced with 5 μM 8-Br-cGMP for 15 minutes. Western blotting was used to analyze microneme content and secretion in the supernatant ESA and pellet. Antibodies α-MIC4 were used for the detection of microneme protein 4 secretion and expression, and α-actin was used as a loading control. **f** Plaque assays revealing growth capabilities of monoclonal parasite line 3# (△*cacnap*-cl.3) and 4# (△*cacnap*-cl.4) of *CACNAP* knockout and parental strains. △*cacnap* exhibited reduced plaques formation compared to parental strain. **g** Statistical analysis of tachyzoite plaque areas. Photoshop was used to calculate the plaque area, and GraphPad Prism 9.0 was used for statistical analysis. Plaques data for each strain were obtained from three independent assays. *$p< 0.05$; **$p< 0.01$; ns, not significant (unpaired two-tailed Student's t test).

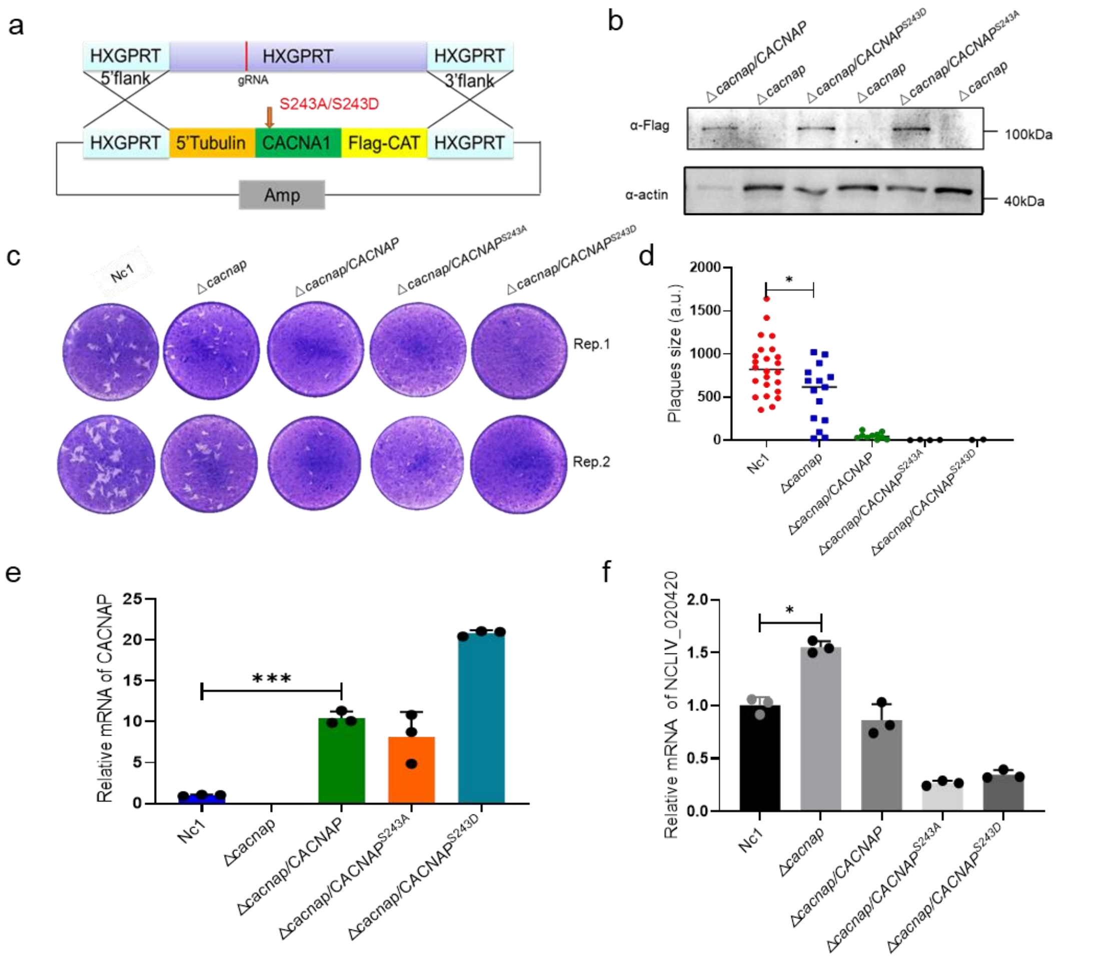


**Supplementary Figure 6 Analysis of growth in different strains regulated by CACNAP gene expression. a** Construction strategy for CACNAP complementation strain, phosphorylated modification site S243A and S243D mutant strains. **b** Western blotting used to detect the expression of CACNAP protein in the gene complementation and phosphorylated modification site S243A and S243D mutant strains. The primary antibody used was α-Flag to detect the Flag tag, and α- actin was used to detect the internal reference protein, actin. **c** Plaque formation in different CACNAP gene-regulated strains**. d** Statistics on the area of plaque formation in different CACNAP-regulated strains. GraphPad Prism 9.0 was used for data statistical analysis. Two-tailed Student's t test was applied to compare the two groups of data.

*$p < 0.05$, ****$p < 0.0001$. **e-f** mRNA expression levels of CACNAP **e** or NCLIV_020420 **f** in different transgenic strains. The relative quantification algorithm (-ΔΔCt) was employed to assess gene expression differences in various CACNAP-regulated strains. ΔCt for each group was determined by subtracting the Ct value of the target gene (Ct target gene) from that of the internal reference (Ct reference gene actin). ΔΔCt was obtained by subtracting the ΔCt of the control group (parental Nc1 strain) from that of the experimental group (gene-regulated strain). The fold difference was calculated using the formula: Fold difference was $2^{-\Delta\Delta Ct}$. Experimental results were presented as mean ± S.E.. One-way ANOVA was conducted using GraphPad Prism 9.0, and pairwise comparison was performed using the Dunnett test. Data show mean values with S.E. from 3 independent assays, *$p < 0.05$, ****$p < 0.0001$, ns, not significant.

# Uncropped gels for Western Blots

## Figure 1c

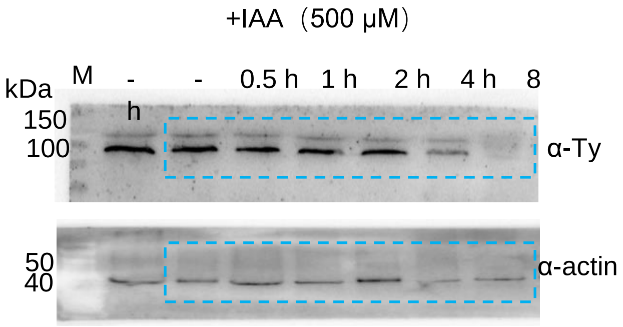


## Figure 2j

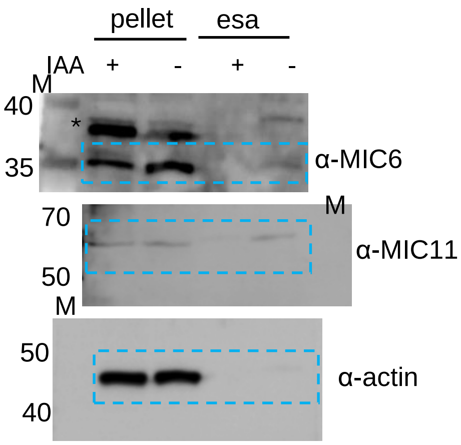


## Supplementary Figure 2i

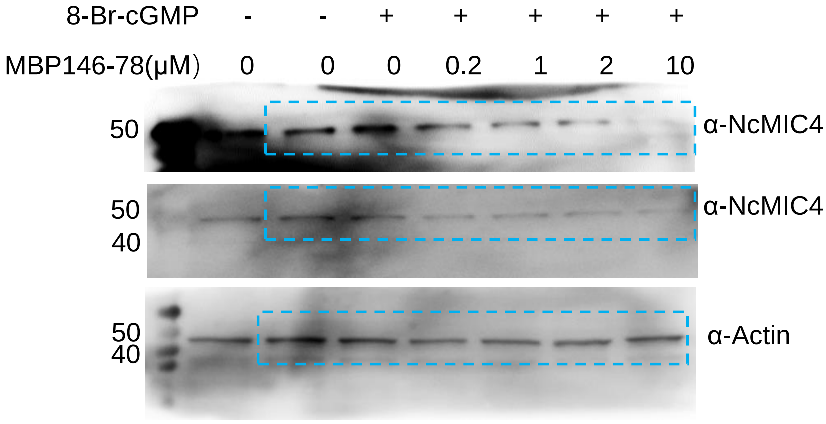


## Supplementary Figure 4e

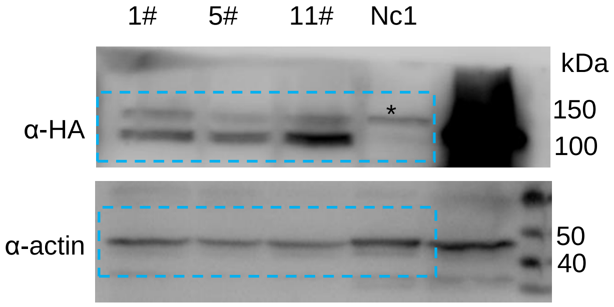


## Supplementary Figure 5e

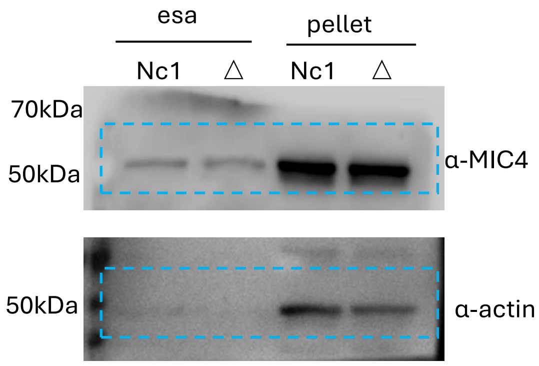

# Uncropped gels for Western Blots

## Supplementary Figure 6b

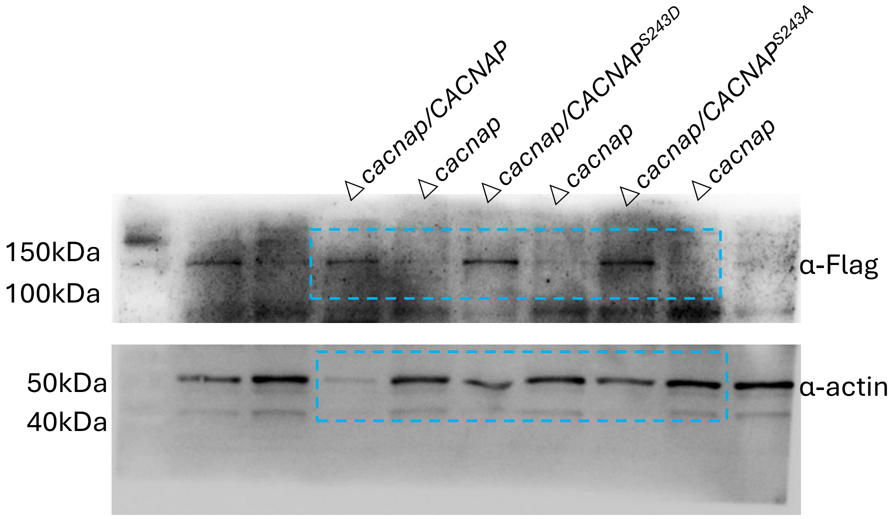

**Supplementary Data 1 Sequence information**

**GeneID inToxoDB: NCLIV_055260**
**Headline: putative AGC kinase TgPKG1**
**Protein Sequence:**
MGACISKNSSARVSRSSALSASKQTVASSAPPGAAGDETSATGAAEEASKKSPARPDGART
SAADLEAAQDASSPEENGQEVGKAELDGARSEKKEDADPRAGMSNSSATSEGDARGDDL
KREVPNQDRSTAEVHESHKEKHGGDRKPAQKAILKQDDCHTEEEKLSAHLAYREKTPAD
FALIRDSLKANLVCSSLNEGEIDALAVAMQFFTFKKGDVVTRQGEPGSYFFIIHSGTFDVLV
NEKRVNAMDKGKAFGEIALIHNTERSATVVASSTEGALWGVQRHTFRETLKQLSSRNFAE
NRQFLASVKFFEMLTEAQKNVITNALVVENFKPGQPIVKEGDAGDVLYILKSGKAKVSIG
GKEIRILRKGDYFGERALLYKEPRSATITAEEFTVCVSIGRELLDRVLGNLQHVLFRNIMVE
ALQQSKVYELFQGDQLSKLIEAAVVKDYAADYVILDKENKTKGIRFFFVLEGELSVYAYV
QNSTTKEEERKLTATLKRGQAFGEEYVLNPTRPFNHYVKSVGPCKLALFTSSVLTATLGGE
DIDETLDFNNKRTIIRKMYIFRYLSDHQMTMLIKAFKTVRYMSGEYIIKEGERGTRFFIIKA
GEVAILKNSKRLRTLGRHDYFGERALLYDEPRTASVCANSAGVDLWVVDKSVFNEIIKGP
MLAHLEERIRMQDTKVEFQDLQVVRVVGRGTFGTVKLVRHVPTNIRYALKCVSRRSVIAL
SQQQHIRLEREIMAENDHPFIIRLVRTFRDKEFLYFLTELVTGGELYDAIRKLGLLARPQAQF
YLASIVLAIEYLHERNIAYRDLKPENILLDNQGYVKLIDFGCAKKMQGRAYTLVGTPHYM
APEVILGKGYTLTADTWAFGVCLYEFMCGPLPFGNDAEDQLEIFRDILTGKLVFPHYVTDQ
DAINLMKRLLCRLPEVRIGCSINGYKDIKEHAFFGDFDWDKLAGRGLAPPLAPKGETYAE
DTEQSSFELDEDDSIVLEDEYDWDKDF*

**GeneID inToxoDB: NCLIV_005460**
**Headline: hypothetical protein**
**Protein Sequence:**
MPGESLSSVPAAVFDAPSSGDTGPLERQEESRFSVPGASASPQSRSPSSGIGDVRESRGVER
VAGPPAASRGQRHDENGGQSSSRSRLAKLLTRLSSGGDQNVHARPSALPQLGAETSGSPPE
SDATHAREDSAASLHAASRVSEGVSSTSVEAPSASGPQTSHIPRLPSQGGGLVSPPSASPPF
ASLETQGSPSEPHPESHLADAFKNRSAVHGHGMGGAYPGRPSRSVESFVVDVRGRWFSEP
SHRRRGQRAEGGSKSRRHRQPRNPAGRTGGDMSVPRGGPALAGTSFAHSVACAVPFVRA
QDGGAALAAGKYVVKHNASKALQALRAVQAEIDRGEQLHNMLGIQKYTEEQKLKELAA
AEPAEDFADQLTCDLKLLRYYTNTEAELELRDRRARAFIYSPLAQIVMAVIIIFNVIFLGFST
LEVSSREYIWDDTQESLKLKRDAPLFVALCSLNCFFALAFFVEMVLRVKCDGLAHFHDFV
NVMDFVNAWFGVADLFIVSIGFFQKVENGVFNSVVALLKLARLLRVLRFIRILKSFLPMRV
LVEGMESSFFSLLYAASFFLIFIYGFAVFFTTAFGFTAEVKEYWGDLFTSMYTLFLILTMTN
WNEIAQRTSVHFTWAKLAIIMYTIFSYFVLFNVVTAVIVEAFSTTAGRLEDEASHFSMFTDF
QVNEQRFESAMAAAQLLRNRRGEVTSLRPSSECSGRRKNVSSSGLRLGAEGNSPPKGEEN
NSCRQQGTPRSVGPVDSTSKGPHQGSFECPRTEAATEERTQPGEGGAFPSKAPGKSAQQR
RTRSRLGSLRTALTIKRRSARQSTAQEATSQRHEDECLLVRSPSFGPTVVSGASGADCSRGP
DADSDGKLQSIAESPHGTESPSLEPFLSSTAAHTLSPPEAERPTERSRVVGEPAREPGALNG
EGPSSFLSSQLSAASREDTASRGFRGQHSWGRSERGWDIASVDDDFPCAWRANSMRRRL
RPNRDDSTFSESKVSLVPNTLDEAFLDVAPDSPYLDLARSHPLQILGERRVQLAMRKCGIS
YIQAYDCLTILVQKGFEEITGQNQLGDDLQNLTPD*

**DNA sequence of plasmid pSAG1-CmR-pTUB1-OsTIR1-Flag:**

CGCGTGTTCTAACCACAAACCTTGAGACGCGTGTTCCAACCACGCACCCTGACACGCG
TGTTCCAACCACGCACCCTGAGACGCGTGTTCTAACCACGCACCCTGAGACGCGTGTT
CTAACCACGCACCCTGAGACGCGTGTTCTGCCGCACAATGTGCACCTGTAGGAAGCTG
TAGTCACTGCTGATTCTCACTGTTCTCGGCAAGGGCCGACGACCGGAGTACAGTTTTT
GTGGGCAGAGCCGTTGTGCAGCTTTCCGTTCTTCTCGGTTGTGTCACATGTGTCATTGT
CGTGTAAACACACGGTTGTATGTCGGTTTCGCTGCACCACTTCATTATTTCTTCTGGTTT
TTTGACGAGT ATGCATGAGAAAAAAATCACTGGATATACCACCGTTGATATATCCCAAT
GGCATCGTAAAGAACATTTTGAGGCATTTCAGTCAGTTGCTCAATGTACCTATAACCAG
ACCGTTCAGCTGGATATTACGGCCTTTTTAAAGACCGTAAAGAAAAATAAGCACAAGT
TTTATCCGGCCTTTATTCACATTCTTGCCCGCCTGATGAATGCTCATCCGGAATTCCGTAT
GGCAATGAAAGACGGTGAGCTGGTGATATGGGATAGTGTTCACCCTTGTTACACCGTTT
TCCATGAGCAAACTGAAACGTTTTCATCGCTCTGGAGTGAATACCACGACGATTTCCG
GCAGTTTCTACACATATATTCGCAAGATGTGGCGTGTTACGGTGAAAACCTGGCCTATT
TCCCTAAAGGGTTTATTGAGAATATGTTTTTCGTCTCAGCCAATCCCTGGGTGAGTTTCA
CCAGTTTTGATTTAAACGTGGCCAATATGGACAACTTCTTCGCCCCCGTTTTCACCATG
GGCAAATATTATACGCAAGGCGACAAGGTGCTGATGCCGCTGGCGATTCAGGTTCATCA
TGCCGTTTGTGATGGCTTCCATGTCGGCAGAATGCTTAATGAATTACAACAGTACTGCG
ATGAGTGGCAGGGCGGGGCTTAA  TTAATCACCGTTGTGCTCACTTCTCAAATCGACAA
AGGAAACACACTTCGTGCAGCATGTGCCCCATTATAAAGAAACTGAGTTGTTCCGTTG
TGGCTTGCAGGTGTCACATCCACAAAAACCGGCCGACTCTAAATAGGAGTGTTTCGCA
GCAAGCAGCGAAAGTTTATGACTGGGTCCGAATCTCTGAACGGATGTGTGGCGGACCT
GGCTGATGTTGATCGCCGTCGACACACGCGCCACATGGGTCAATACACAAGACAGCTA
TCAGTTGTTTTAGTCGAACCGGTTAACACAATTCTTG CCCCCCCGAGGGGGATCCACT
AGTTCTAGAGCGGCCTCGAGGTCGACGGTATCGATAAGCTAGAGCTTCAGCATCATCTC
TGGAAGCATCCCCTGAACTGCCTGAGTCTACCAAGAGCACTGGCGAAGGCTGTGAGT
AGTCGGACAGGCACGGTGACTCATGTTGTTGGACAGTAGCGAGCTCTGGGTTAACCGC
ATATTCACTAACTGGCTCCGTCCTGTTGTCATTAGATACTGAATCAGGTAACGATACATG
AGCAGCATCCTCGTGTTCCAGGCGCATGTCCTGCTCCGGCTTGCAACCAAGGACCCGT
GGTTCATCTTTGGGTCTCTCCGTACTGGGTGGTAGAGGTGAAACTGTCGACGTGGATG
CAGCTGCCCTGCTTAGAGTACGGACGAAGTGAATAGCTGCGTCTGCATGAACAAGGGG
CTCTGAGGCCCGCTGTGATACGAAAGGTTTGCTGGCTACTGAACATAGGTCTCGCAGT
GCGGGGGCATACTCCAGTCGGCCTTCACGAAACTTCGTGACCAGGCGATCAACAAGC
GGCGTCACGCGAGTTAAATCCGTCCAGAGAAAGCCACCATAGTGCACCATATACTGCC
GGCACATCTTGCTGAAAGTCGTGGCGTGTCGATCAGCTAAGTCCTGTGTGTACTTGGA
AGTGAGCAGCTCTTCCAGTTCGGTCCAGTCATATTGCCATGCCCTAGCCACCGTTTCAC
CAGCGTCCGATGCAGGCTCACCTAGCCGATACCTGTGACCGTTCATATATGAGTTGCTAT
TACATCTGTCGGCCGTAACGACACAAGGAGATGCGGCTGGAGGCAACGGGTTTGTGA
GCACCATTTTGACTCTGTCACGGAGTAAAAAACATTTATTCGAACTTTGTACGAGCGCA
GTCAGTAGAAGTCAACCACGCGTATCAACTACGCTGCAATTACAGAGGACAGGGACA
GGGAAAAAAAGCCGAAGAGGTTGCCGGTGTTAGGAGATGACGAGACGTTTAAACGGC
CGCTGGCTATTGTTCGGGCATCGTTGAGCCACAAGCGATCAAGGTGAAAACAAAGTTA
AATAGTTATGCTGGAGCGATTGCCCTGCTGAATCTTCAGATCGGACGAACAGTCATTCT

GGCCCACTGTACTTGATGTGTTCGATGTAAATCACACTCAGTCGCGTGCTCGGTAGCAA
TCAAGTTGCTCTTTTCTCTCCTTTCTAGACACGGTAAGAACGCTTATGAACACGCATAC
ACGCATAGTTTTTGCTAGAATGCAGCGACCAGATGTCGCAAGGTCGTCTCCCCCATCGA
CTGGAGAATCAAGAAAAACCTGCGTTGATCCCAAACGTACTCTGTGGTTGGTGCAACC
AGAAGTTTCATACTGATCAAAAGCCAGTGACAGCTGGGGACATTGCAGGTCTGGTGCT
TCAAGAAGCGCTGAAGAAGAAAGTGGCGAAACCCTCGGCAGTGCCTTGAAAAGAGG
CGCCGTGCATGTAACTTTTGAAGTGCGTAGTACCTGGCTCCTAATGCTGTTTTGTGTTTC
GCTGTCTGGGCAGCAGTAGAATGCTGTGCCAGAATTAGCCACTATTTTAGACATTTATTT
ACACATTTTTTTTCTGATGAACTTGGCTTATTCATTTTTTCAAGTCTTGCCACTGGGTGG
TGGCATGAGACTCGCTTAGATGTATGTGGGTGTTGCAATCACGCTGATGCTCGGCTTAT
TTCTGAGTTTTTTGTGGTTTTGACAATGGGAACGATTTCAGAGCTACTATTTCACGTGG
TACGGTTATGAGCCACTAAAAAAACGAAGAAAAACGCTGTTTGCAGAAACAATAGCA
AACTGTTTTTCGTCATAGTAACTCAGCGCCCCCTTGCCCCCCCCCAGCAGTGAGATGCA
AGACAATCCTCTCTACCACAGCTTTTGTTGCGTCTGTTTCAAATTTTCAGCGCTCGCGA
AAGGCATCACGAACAACATTATGAGAGGGAGCAGGTTTGTGGGGCTGGCGGGTGCAG
GAATGTGTTCCTGCGAAAAAGGCCTCTGCTGAGAAGGTCGTGGCGTTTGAAAAATATC
CGAGGTAGCAAAGACTTGTTTCAGTGCTCCCCTTTTGAAGACCTGCGGCGGCAGTGCA
CTGAAGAGTAACTCCAAATCACCGCGGTGAGACTTGGTTTTTTCCGTTATCCTTCAGAA
GAGTGTGTTTTCGTTTAATTCGTCACAGACCACGAAAAACGAACCATCGAAGACGATC
ACTGCGTCCGCGTGCATCTGGATGGATGACCCACATCTGTTGCAGCCGTCGCAGACAT
GCATGTCCCGCGTTCGTGAAATTCTCTGCATCAGCGGAGTGATCAGGAATCATCGTCTC
AGCGGGATGACGTTGCGGAGCAGGCCGGCTCGCGGTGGGCAGTCAGATGCCGAAGGC
GTAACTCAGGACGGCTTGCGCTCATCGCAGAACAGGGGTGGTGCCTGCATTGGGTGCG
GTTGGTGATCCTGGTTGGACCGGTGGAGATGCGCGCGCACGAAGGGGATGTGTCAGA
AACATTTTGTTTGTTCTCTGTGAACTTTTAGATGTGTTAAAGGCGGCGAATATTAGCAG
AGAGTCCTCCTTGTTGGATTCTCTCTTGAATTTCGCCCTTTCCTTCTCTTTGCGAGTCTC
GTAGAGAACAAGCACTCGTTCGCCGTCCCTGACGACGCAACCCGCGCAGAAGACATC
CACCAAACGGTGTTACACAATCACCTTGTGTGAAGTTCTTGCGGAAAACTACTCGTTG
GCATTTTTTCTTGAATTCCCTTTT AGATCTAAAATGACCTACTTCCCTGAGGAAGTGGT
CGAGCACATTTTTTCTTTCCTCCCCGCACAAAGGGACAGGAACACGGTTAGTCTGGTC
TGCAAGGTGTGGTACGAGATCGAACGCCTTAGCCGCCGAGGTGTTTTCGTCGGAAATT
GCTACGCTGTGCGTGCCGGTCGGGTCGCCGCCCGATTTCCCAATGTCCGAGCGCTCAC
CGTGAAAGGAAAGCCACACTTTGCGGATTTTAATCTCGTGCCTCCCGACTGGGGTGGG
TACGCTGGCCCCTGGATCGAAGCTGCAGCGAGGGGATGTCACGGCCTCGAAGAACTG
CGTATGAAAAGAATGGTCGTTAGCGACGAAAGCCTGGAACTTTTGGCACGTTCGTTTC
CACGCTTCCGCGCGCTGGTCCTTATCTCCTGCGAAGGATTCTCTACGGACGGTTTGGCA
GCGGTCGCCTCTCATTGTAAACTCTTGCGAGAGCTCGATCTGCAGGAGAACGAGGTGG
AGGATCGAGGACCTCGCTGGCTGTCTTGCTTCCCGGACAGTTGTACTAGCCTTGTGAG
CCTGAACTTCGCATGTATTAAGGGTGAAGTGAACGCCGGCTCCCTGGAGCGCTTGGTG
TCCCGCTCCCCCAACCTTCGTTCTCTCAGACTGAACAGAAGCGTCAGCGTTGACACCC
TTGCCAAGATCCTGCTGCGAACGCCGAACCTCGAGGACTTGGGCACAGGCAATCTCAC
TGATGACTTCCAAACCGAGTCGTATTTCAAGCTGACATCTGCTTTGGAAAAGTGTAAG
ATGCTCCGGAGCCTGAGTGGCTTTTGGGATGCAAGCCCGGTCTGCCTCTCGTTCATCTA
CCCCTTGTGTGCGCAACTGACAGGCCTCAATCTTTCGTATGCCCCGACATTGGATGCCT

CTGACCTCACCAAAATGATCTCCAGATGTGTTAAGCTCCAACGGCTCTGGGTGCTTGAT
TGCATTAGCGACAAGGGCCTGCAAGTGGTCGCGTCCTCTTGCAAGGATCTGCAGGAGC
TGCGCGTGTTTCCATCTGACTTCTATGTCGCCGGATATAGTGCAGTCACGGAAGAGGGA
CTCGTGGCGGTCTCTCTGGGCTGCCCTAAGCTTAACTCTCTCCTCTACTTCTGCCATCA
GATGACCAACGCCGCGCTCGTGACGGTCGCGAAGAACTGCCCCAACTTCACCCGCTTT
AGACTCTGTATCCTTGAACCAGGGAAGCCCGACGTGGTCACGTCGCAGCCTCTCGATG
AGGGCTTTGGCGCGATCGTTCGGGAATGCAAGGGTTTGCAACGTTTGTCGATTAGCGG
GCTTTTGACGGATAAGGTTTTTATGTACATCGGAAAGTACGCCAAGCAGCTCGAAATGC
TGTCCATTGCTTTCGCCGGGGACAGCGACAAGGGGATGATGCATGTTATGAATGGATGC
AAGAACCTGAGAAAGCTGGAGATCCGCGATTCGCCGTTCGGCGATGCGGCCCTCCTGG
GAAACTTCGCCCGATACGAGACTATGCGTTCTCTCTGGATGTCGAGCTGTAACGTCACG
CTTAAAGGTTGCCAAGTTCTTGCAAGCAAGATGCCCATGTTGAACGTGGAAGTGATTA
ACGAGAGGGACGGATCTAACGAGATGGAGGAGAACCACGGCGACCTCCCAAAGGTG
GAAAAGCTTTACGTCTACAGGACAACTGCAGGAGCGAGAGACGATGCCCCAAACTTT
GTCAAAATCCTG ggtggaggtggaagt GACTATAAGGACCACGACGGAGACTACAAGGATCA
TGATATTGATTACAAAGACGATGACGATAAG TAAATGCATGCCACAGAAGCTGCCCGT
CTCTCGTTTTCCTCTCTTTTCGGAGGGATCAGGGAGAGTGCCTCGGGTCGGAGAGAGC
TGACGAGGGGGTGCCAGAGACCCCTGTGTCCTTTATCGAAGAAAAGGGATGACTCTTC
ATGTGGCATTTCACACAGTCTCACCTCGCCTTGTTTTCTTTTTGTCAATCAGAACGAAA
GCGAGTTGCGGGTGACGCAGATGTGCGTGTATCCACTCGGAATGCGTTATCGTTCTGTA
TGCCGCTAGAGTGCTGGACTGTTGCTGTCTGCCCACGACAGCAGACAACTTTCCTTCT
ATGCACTTGCAGGATGGTGCAGCGCAAACGACGGAGAGAAAGGAGCACCCTCTCAGT
TTCCCTACGATGTGCTGTCAGTTTCGACTCTTCACCGCGAACGATTGGCGATACGTCTC
TGTTGACTTGTTAGGCTCCGACCACGAAGCTCCCTTAACTAGATAAGCCGCGACACCTA
AGTGTACACCATTTGCAGATCGATAATCTGCGACCGCTGAATCCGTCCAGATCAGTAAA
ACCGCACCACCTAAGTGTAAACCTTGTTTAGGTCGATAAAATGCTACCAACCCCCACCC
ACAATCGAGCCTTGAGCGTTTCTGCGCACGCGTTGGCCTACGTGACTTGCTGATGCCT
GCCTCTGGCCATTCATGCCAGTCAGTGCGCATAAAAATGTGGACACAGTCGGTTGACA
AGTGTTCTGGCAGGCTACAGTGACAC
