# Supplementary material for: Unveiling the impact of cGMP-dependent protein kinase of Neospora caninum on calcium fluxes and egress functions through quantitative phosphoproteome analysis
Source: Commun Biol. 2025 May 13;8:744. doi: 10.1038/s42003-025-08173-x (PMC12075863; doi:10.1038/s42003-025-08173-x)
Supplement: Supplementary file 3 — Description of Additional Supplementary Materials [file 42003_2025_8173_MOESM3_ESM.pdf]

Description of Additional Supplementary Files

File name: Supplementary Data 1

Description: Sequence information

File name: Supplementary Data 2

Description: The source data behind the graphs in the paper

File name: Supplementary Data 3

Description: The plasmids and primers listed in the Materials and methods.

File name: Supplementary Table 1

Description: Phosphorylation downstream protein information.Summary of the phosphoproteomic analysis of the N. caninum tachyzoites treatment(MBP group) or untreatment(Vehicle group) with PKG inhibitors MBP164-78.
