# Supplementary material for: Unveiling the impact of cGMP-dependent protein kinase of Neospora caninum on calcium fluxes and egress functions through quantitative phosphoproteome analysis
Source: Commun Biol. 2025 May 13;8:744. doi: 10.1038/s42003-025-08173-x (PMC12075863; doi:10.1038/s42003-025-08173-x)
Supplement: Supplementary file 8 — Reporting summary [file 42003_2025_8173_MOESM8_ESM.pdf]

nature portfolio

Corresponding author(s): Jing Liu

Last updated by author(s): Apr 29, 2025

Reporting Summary

Nature Portfolio wishes to improve the reproducibility of the work that we publish. This form provides structure for consistency and transparency in reporting. For further information on Nature Portfolio policies, see our Editorial Policies and the Editorial Policy Checklist.

Statistics

For all statistical analyses, confirm that the following items are present in the figure legend, table legend, main text, or Methods section.

n/a	Confirmed	
☐	☒	The exact sample size (*n*) for each experimental group/condition, given as a discrete number and unit of measurement
☐	☒	A statement on whether measurements were taken from distinct samples or whether the same sample was measured repeatedly
☒	☐	The statistical test(s) used AND whether they are one- or two-sided<br>*Only common tests should be described solely by name; describe more complex techniques in the Methods section.*
☒	☐	A description of all covariates tested
☒	☐	A description of any assumptions or corrections, such as tests of normality and adjustment for multiple comparisons
☒	☐	A full description of the statistical parameters including central tendency (e.g. means) or other basic estimates (e.g. regression coefficient) AND variation (e.g. standard deviation) or associated estimates of uncertainty (e.g. confidence intervals)
☐	☒	For null hypothesis testing, the test statistic (e.g. *F*, *t*, *r*) with confidence intervals, effect sizes, degrees of freedom and *P* value noted<br>*Give P values as exact values whenever suitable.*
☒	☐	For Bayesian analysis, information on the choice of priors and Markov chain Monte Carlo settings
☒	☐	For hierarchical and complex designs, identification of the appropriate level for tests and full reporting of outcomes
☒	☐	Estimates of effect sizes (e.g. Cohen's *d*, Pearson's *r*), indicating how they were calculated

Our web collection on statistics for biologists contains articles on many of the points above.

Software and code

Policy information about availability of computer code

Data collection	no
Data analysis	no

For manuscripts utilizing custom algorithms or software that are central to the research but not yet described in published literature, software must be made available to editors and reviewers. We strongly encourage code deposition in a community repository (e.g. GitHub). See the Nature Portfolio guidelines for submitting code & software for further information.

Data

Policy information about availability of data

All manuscripts must include a data availability statement. This statement should provide the following information, where applicable:

- Accession codes, unique identifiers, or web links for publicly available datasets
- A description of any restrictions on data availability
- For clinical datasets or third party data, please ensure that the statement adheres to our policy

Data supporting the conclusions of this article are included within the manuscript and its supplementary files. Source data for all graphs and plots in the paper can be found in files Supplementary Data 2. The phosphoproteomics data are available via the ProteomeXchange Consortium (https://proteomecentral.proteomexchange.org) and the iProX database (https://www.iprox.cn/) with the dataset identifier PXD062765 and IPX0011618000. The datasets generated and analyzed during the current study are available from the corresponding authors upon reasonable request.

Research involving human participants, their data, or biological material

Policy information about studies with human participants or human data. See also policy information about sex, gender (identity/presentation), and sexual orientation and race, ethnicity and racism.

Reporting on sex and gender	no
Reporting on race, ethnicity, or other socially relevant groupings	no
Population characteristics	no
Recruitment	no
Ethics oversight	no

Note that full information on the approval of the study protocol must also be provided in the manuscript.

Field-specific reporting

Please select the one below that is the best fit for your research. If you are not sure, read the appropriate sections before making your selection.

☒ Life sciences ☐ Behavioural & social sciences ☐ Ecological, evolutionary & environmental sciences

For a reference copy of the document with all sections, see nature.com/documents/nr-reporting-summary-flat.pdf

Life sciences study design

All studies must disclose on these points even when the disclosure is negative.

Sample size	The sample size was estimated by using the experimental methods described in the published article and determined by a pre-experiment. The sample size was obtained by averaging three repeated cell counts
Data exclusions	No data were excluded from the analysis
Replication	Representative data has been repeated a minimum of two times, each with at least two independent biological samples and three technical replicates. The results were consistent with the replicates
Randomization	Random distribution of samples
Blinding	no blinding

Reporting for specific materials, systems and methods

We require information from authors about some types of materials, experimental systems and methods used in many studies. Here, indicate whether each material, system or method listed is relevant to your study. If you are not sure if a list item applies to your research, read the appropriate section before selecting a response.

Materials & experimental systems

n/a	Involved in the study	
☐	☒	Antibodies
☒	☐	Eukaryotic cell lines
☒	☐	Palaeontology and archaeology
☐	☒	Animals and other organisms
☒	☐	Clinical data
☒	☐	Dual use research of concern
☒	☐	Plants

Methods

n/a	Involved in the study	
☒	☐	ChIP-seq
☒	☐	Flow cytometry
☒	☐	MRI-based neuroimaging

Antibodies

Antibodies used	Commercially available antibodies, including as mouse or rabbit anti- hemagglutinin (HA), mouse or rabbit anti-Flag, were obtained from Thermo Fisher Scientific. FITC/Cy3 labeled sheep anti-rabbit/mouse IgG and HRP labeled sheep anti-rabbit/mouse IgG

	purchased from M&C Gene Technology, Beijing. Additionally, antibodies like mouse anti-Ty, mouse anti-NcMIC4, mouse anti-NcMIC6, mouse anti-NcMIC11, mouse anti-actin, rabbit anti-NcSRS2, were generated before, preserved in China Agricultural University, Beijing and used in our study.
Validation	Relevant citations for antibodies have been provided in the manuscript

Animals and other research organisms

Policy information about studies involving animals; ARRIVE guidelines recommended for reporting animal research, and Sex and Gender in Research

Laboratory animals	Five-week-old female BALB/C mice
Wild animals	No wild animals
Reporting on sex	The results were not influenced by gender
Field-collected samples	The study did not involve samples collected from the field
Ethics oversight	All animal procedures and experiments performed in this study were approved by the Laboratory Animal Welfare and Animal Experimental Ethical Committee of China Agricultural University (Certificate No. CAU-AW31901202-2-1).

Note that full information on the approval of the study protocol must also be provided in the manuscript.

Plants

Seed stocks	no
Novel plant genotypes	no
Authentication	no
